# Multiplexed Analysis of Cage and Cage Free Chicken Egg Fatty Acids Using Stable Isotope Labeling and Mass Spectrometry

**DOI:** 10.3390/molecules181214977

**Published:** 2013-12-05

**Authors:** Richard G. Torde, Andrew J. Therrien, Michael R. Shortreed, Lloyd M. Smith, Shane M. Lamos

**Affiliations:** 1 Department of Chemistry, University of Vermont, 82 University Place, Burlington, VT 05405, USA; E-Mail: richard.torde@uvm.edu; 2 Department of Chemistry, Tufts University, 62 Talbot Ave., Medford, MA 02155, USA; E-Mail: andrew.therrien@tufts.edu; 3 Department of Chemistry, University of Wisconsin, 1101 University Avenue, Madison, WI 53706, USA; E-Mails: mshort@chem.wisc.edu (M.R.S.); smith@chem.wisc.edu (L.M.S.); 4 Department of Chemistry and Physics, Saint Michael’s College, 1 Winooski Park, Colchester, VT 05439, USA

**Keywords:** fatty acids, cholamine, pre-ionization charge, stable isotope labeling, multiplexing

## Abstract

Binary stable isotope labeling couple with LC-ESI-MS has been used as a powerful non-targeted approach for the relative quantification of lipids, amino acids, and many other important metabolite classes. A multiplexed approach using three or more isotopic labeling reagents greatly reduces analytical run-time while maintaining excellent sensitivity and reproducibility. Three isotopic cholamine labeling reagents have been developed to take advantage of the pre-ionized character of cholamine, for ESI, and the ease by which stable isotopes can be incorporated into the cholamine structure. These three cholamine labeling reagents have been used to relatively quantify three fatty acid samples simultaneously. The quantification resulted in the observation of 12 fatty acids that had an average absolute error of 0.9% and an average coefficient of variation of 6.1%. Caged *versus* cage-free isotope labeling experiments showed that cage-free eggs have an increased level of omega-3 fatty acids as compared to caged eggs. This multiplexed fatty acid analysis provides an inexpensive and expedited tool for broad-based lipid profiling that will further aid discoveries in the mechanisms of fatty acid action in cells.

## 1. Introduction

Comprehensive quantitative analysis of the metabolome is a critical step in a systems biology approach to understanding metabolic response to external stimuli [1]. Such metabolomic approaches are varied in their analytical nature due to the vast chemical space occupied by the metabolome. The high resolving power of mass spectrometry (MS) coupled with chromatography, liquid chromatography (LC) or gas chromatography (GC) has been most often employed for reducing the chemical complexity and exacting the individual identity of members within a metabolomic sample [2,3,4,5,6]. One way that absolute quantification of individual metabolites has been performed is by using structural analogs as internal standards with data analysis software that corrects for differences in ionization properties between the metabolite of interest and the structural analog [7,8,9]. Stable isotope dilution (SID) is another more targeted approach to quantitative metabolite analysis that relies on isotopic analogs of metabolites of interest as internal standards. These isotopic analogs provide for greater precision and accuracy than structural analogs in metabolite quantification due to their ability to co-elute with the naturally occurring metabolite, thus mitigating differential ion suppression that can arise from chromatographic retention time differences between the analog and the metabolite of interest [10]. SID has been used in some exemplary *in vivo* [11,12] approaches as well as a number of *ex vivo* approaches [13,14]. While very powerful as a metabolomics approach, SID is limited by a number of technical challenges.

(1) SID can only be used to quantify metabolites where a commercially available isotopic analog exists.
(2) The isotopic analogs should not contain deuterium, since such analogs will exhibit a small chromatographic separation from the endogenous protium from which can lead to differential suppression of ionization.
(3) Many common metabolites do not sufficiently ionize in conventional MS, with or without isotopic atoms present, and thus are not quantified in a SID approach.

An alternative approach to SID employs isotopic labeling reagents that target prevalent functional groups of metabolites [15,16,17,18,19]. The benefits of such a chemical tagging approach include; the ability to incorporate isotopes into molecules for which no commercially available isotopic analog exists, provide an inexpensive source of isotopic incorporation that does not impact chromatographic resolution, the realization that a tagged molecule indicates that a metabolite contains a certain functional group-the one targeted by the reagent, and, if designed properly, the chemical label can enhance ionization and thus sensitivity in a MS metabolite analysis. Our group and others have previously demonstrated the utility of such an isotopic labeling reagent approach wherein a control and experimental sample are labeled with a reagent that differs only in its isotopic composition [16,20]. Relative quantification of metabolites between the two samples has helped researchers unravel some of the complexity in biological systems by elucidating differences in metabolite levels [21,22].

Despite a large number of binary isotopic labeling methodologies there remain a limited number of multiplexed isotopic labeling strategies wherein three or more isotopically unique labeling reagents are used to label and quantify three or more samples simultaneously. Multiplexed approaches in the “-omics” fields provide the opportunity for higher throughput analyses. Indeed, several multiplex approaches have even found commercial success in the proteomics field [23,24,25]. An increased understanding of metabolites can also be realized through new multiplexed metabolomic labeling strategies. The purpose of this study was to demonstrate a multiplexed approach for fatty acid and other carboxylic-acid containing metabolites and to investigate the differences in fatty acid composition among “caged” and “cage free” chicken eggs. This multiplexed tool should afford opportunities for expedited the analyses of other complex carboxylic acid-containing metabolite samples.

## 2. Results and Discussion

### 2.1. Synthesis and Evaluation of the Three Isotopic Labeling Cholamines

**Scheme 1:**
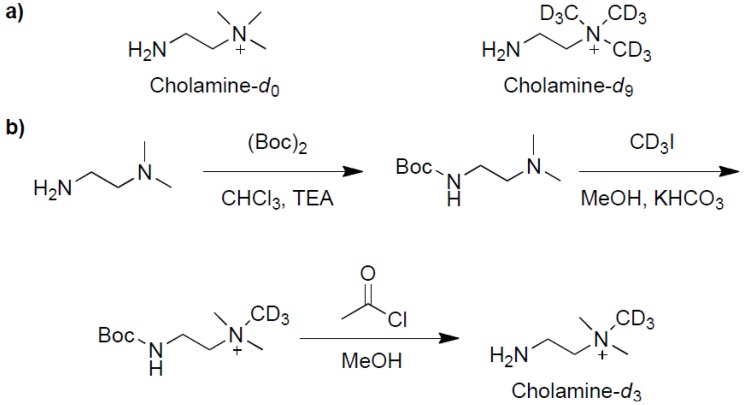
(**a**) Structures of cholamine-*d_0_* and cholamine-*d_9_*. (**b**) Synthesis of cholamine-*d_3_*, from N,N-dimethylethylenediamine (reagents: TEA: triethylamine; (Boc)_2_: di-*tert*-butyl dicarbonate; Boc: *tert*-butoxycarbonyl; MeOH: methanol).

**Scheme 2:**
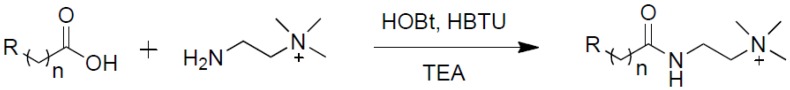
Fatty acid labeling with isotopic cholamines gives improved electrospray ionization and allows for relative quantification (reagents: HOBt: hydroxybenzotriazole; HBTU: N,N,N′,N′-tetramethyl-*O*-(1*H*-benzotriazol-1-yl)uronium hexafluorophosphate).

Our multiplex derivatization strategy utilizes cholamine in three synthetic isotopic forms (*d_0_*, *d_3_*, *d_9_*—the notation *d_x_* is used to indicate that the compound contains x deuterium atoms) as the labeling reagent for fatty acids. We have reported the synthesis of cholamine-*d*_0_, “light”, and cholamine-*d*_9_, “heavy” previously (Scheme 1a) [16]. The synthesis of our new cholamine reagent, cholamine-*d*_3_, “medium”, is shown in Scheme 1b. Isotopic labels are incorporated as deuterium atoms through the methylation step in the synthesis of cholamine-*d*_3_. The cholamine labeling reagents convert carboxylic acid-containing metabolites into an amide bond through a coupling reaction (Scheme 2). We have chosen the cholamine labeling reagent for our multiplexed approach because of several advantages it provides in LC-MS based metabolite quantification experiments [16]. The quaternary ammonium group gives the labeled compound a pre-ionization charge, greatly enhancing detection by positive mode ESI-MS [26,27].

We observe limits of detection for cholamine-labeled fatty acids (15–30 fmol) that are 1.5 to 3 times lower than the LOD’s for fatty acids analyzed under their optimal negative-mode conditions. Separation in the LC is maintained for all labeled fatty acids due to the small size of the cholamine label relative to the target analytes. We also observe co-elution of labeled isotopic triplets, as reported previously in binary systems [16,28], for deuterium incorporation on the quaternary ammonium functional group as opposed to the chromatographic shift problems that can arise when the deuterium isotopes are incorporated at more hydrophobic positions [17].

### 2.2. Simulated Multiplex Quantification of Lauric Acid

**Figure 1:**
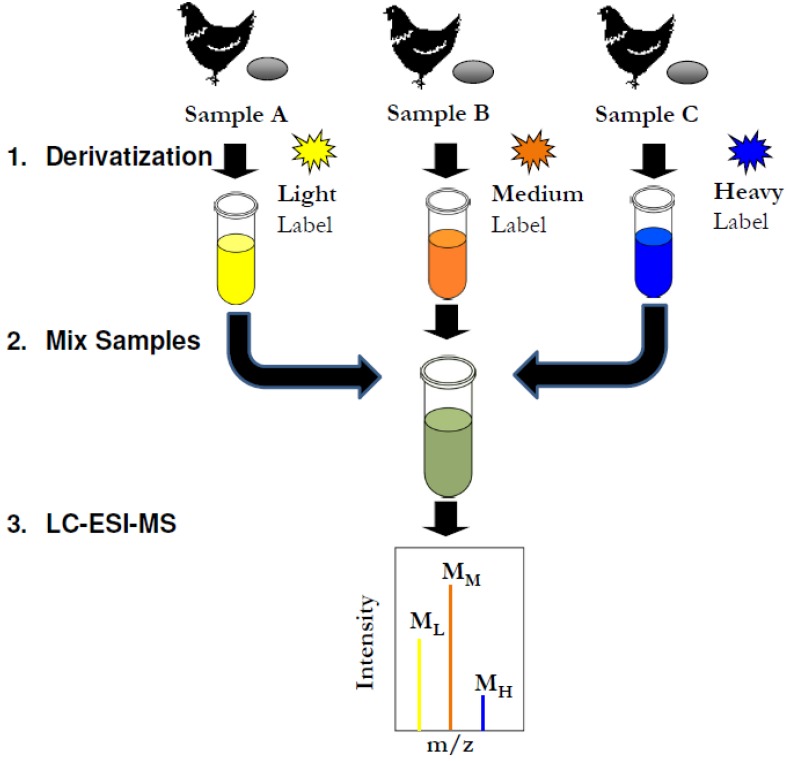
Multiplex relative quantification with isotopic labeling. Fatty Acids are extracted from three different egg samples before labeling of each with a unique form of cholamine (light, medium, or heavy). The resulting isotopically labeled fatty acids are combined and analyzed by LC-ESI-MS wherein relative quantification can be made from the peak intensities of the fatty acids from the individual sample.

A multiplex analysis allows for the labeling of three samples each with a reagent that differs only in its isotopic composition, thereby creating “heavy”, “medium”, and “light” versions of derivatized metabolites, which are easily distinguished by MS (Figure 1).

Three identical samples of lauric acid were prepared and reacted with either *d_0_-*, *d_3_-*, or *d_9_-*cholamine as described in the Experimental section. The three labeled solutions were subsequently mixed in ratios of 1:1:1 to simulate relative quantification for a single fatty acid. Extracted ion chromatograms (EIC’s) were obtained for the masses corresponding to the light-, medium-, and heavy-labeled version of lauric acid from three analytical runs. The average experimental ratio for lauric acid was 0.99:0.99:1.01 *versus* an expected ratio of 1:1:1. The accuracy and precision were very good for this simulated relative quantification experiment: average absolute error was 0.9% and the average coefficient of variation (CV) was 6.1%.

### 2.3. Simulated Multiplex Fatty Acid Analysis

Fatty acids were extracted from a commercial egg yolk as described in the Experimental section. Three identical fatty acid samples were produced from this single extract and were each reacted with one of the three cholamine labeling reagents (*d_0_-*, *d_3_-*, or *d_9_-*cholamine). The three labeled extracts were subsequently mixed in a ratio of 1:1:1. The mixture was analyzed by LC-ESI-MS and EIC’s were obtained for 10 cholamine labeled fatty acids. The ratios of peak intensities were calculated for each triplet grouping of light, medium, and heavy-labeled fatty acids. The experimental ratios for the 10 fatty acids are displayed in Table 1. The results showed an average experimental ratio of 0.97:1.01:1.01 with an average absolute error of 5.4%.

**Table 1:** Simulated multiplexed fatty acid analysis where the expected ratio of fatty acids was 1.00:1.00:1.00, *d_0_*:*d_3_*:*d_9_*.

Fatty acid ^a^		Experimental ratio			Accuracy
		*d_0_*	*d_3_*	*d_9_*	(% error)
Myrisitc acid	14:0	1.01	1.01	0.98	1.9
Myristoleic acid	14:1	1.03	0.95	1.02	4.1
Palmitic acid	16:0	0.98	1.03	0.99	2.7
Palmitoleic acid	16:1	0.95	0.96	1.09	8.0
Stearic acid	18:0	0.97	1.02	1.00	2.4
Oleic acid	18:1	1.08	0.95	0.96	7.2
linoleic acid	18:2	0.96	1.00	1.04	3.7
α-linolenic acid	18:3	1.04	0.93	1.03	6.4
Eicosatrienoic acid	20:3	0.90	1.10	1.00	10.4
Arachidonic acid	20:4	0.90	1.10	1.00	9.6
Docosapentaenoic acid	22:5	0.93	1.05	1.02	6.0
Docosahexaenoic acid	22:6	0.99	1.02	0.99	2.1
Average		0.97	1.01	1.01	5.4

^a^ Fatty acids are referred to by their number of carbons and degree of unsaturation.

### 2.4. Multiplex Quantification of Fatty Acid Content in Three Different Commercial Egg Sources

**Figure 2:**
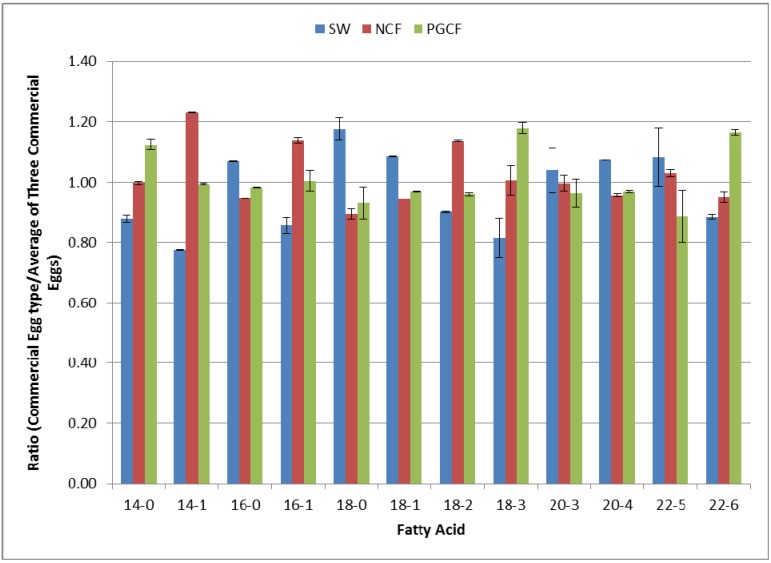
Multiplexed quantification of fatty acids in hydrolyzed egg lipid extracts from caged and cage free chickens. The results of quantification are displayed as ratios between the commercial sample and the average of the three commercial samples.

Multiplexed stable isotope labeling was used to analyze egg fatty acid profiles from three different commercial eggs as shown in Figure 2. The fatty acids from all three samples were extracted separately as described in the Experimental section. Each of the three fatty acids extracts were labeled with the following cholamines: Shaw’s White Eggs (SW)-cholamine-*d_0_*, Nellie’s Cage Free Brown Eggs (NCF)-cholamine-*d_3_*, and Pete & Gerry’s Cage Free Ameraucana (blue) Eggs (PGCF)-cholamine-*d_9_*. The ratios in Figure 2 correspond to the content of the fatty acid in an individual sample as compared to the average amount of that fatty acid in all three samples. A value of 1 in Figure 2 indicates a fatty acid sample that is in average abundance as compared to all three experimental eggs. The error bars in Figure 2 represent the standard deviations obtained from three multiplexed LC-MS runs. The mean CV across the 12 different fatty acids was 2.1% (range of 0.1%–6.6%). This level of precision is similar to that reported for other metabolite profiling approaches that employ silylation labeling with GC-MS (mean CV range 8.2%–12.6%) [29,30] or LC-MS (mean CV range 15%–16%) [31,32] without the use of an isotopic labeling reagent.

### 2.5. Discussion

Our multiplexed approach was demonstrated on a single fatty acid, lauric acid, as well as on a fatty acid extract from egg yolk. In each case, the quantification yielded low absolute errors (<10%) and low coefficient of variations (<10%) (Table 1). The precision of these analyses is quite similar to our previously reported values for a binary mixture as well as for those reported in prior GC/MS and LC/MS based metabolite experiments.

Fatty acid composition of dietary fat has been implicated in a number of health related pathways. Modern chicken farming approaches have been developed to influence the fatty acid composition of chicken eggs [33,34]. It has been observed that chickens fed a diet high in vegetable greens and insects, “cage free”, lead to higher levels of omega-3 fatty acids, such as docosahexaenoic acid, being incorporated into the resulting eggs [35]. Our multiplexed cholamine labeling was used for the relative quantification for fatty acids in eggs lipids in order to investigate the dietary fat present in three different commercially available chicken eggs. As seen in Figure 2, both α-linolenic acid (18:3) and docosahexaenoic acid (22:6) showed increased levels in both types of cage free eggs (NCF and PGCF) as compared to the commercial Shaw’s white egg (SW). This increase in omega-3 fatty acids is consistent with the observation that cage free produced eggs result in higher levels of omega-3 fatty acids [36]. This multiplexed approach to fatty acid analysis has the potential to significantly enhance the detection and quantification of these important fatty acids as biomarkers. In the future, we plan to extend the scope of our multiplexed stable isotope labeling approach by targeting other important classes of metabolites. This goal will be realized through the development of new labeling reagents that will target a broad range of functional groups.

## 3. Experimental

### 3.1. General Information

^1^H-NMR spectra were measured on a Varian Gemini-300 instrument (300 MHz, Varian, Palo Alto, CA, USA). Mass spectra were recorded on an electrospray ionization (ESI) FT/ion-trap mass spectrometer (LTQ Orbitrap Velos, Thermo Fisher Scientific, San Jose, CA, USA). All chemicals were purchased from Sigma-Aldrich (Milwaukee, WI, USA) unless otherwise noted. (2-Aminoethyl)trimethylammonium chloride hydrochlorides (cholamine-*d_0_* and -*d_9_*) were prepared as we have previously described [16]. Cage and cage-free eggs were obtained from Shaw’s (Spokane, WA, USA) and stored at 0 °C until used.

### 3.2. Synthesis of Cholamine-d_3_

Cholamine-*d_3_* was prepared starting from commercially available *N*,*N*-dimethylethylenediamine (Scheme 1b). The *N*,*N*-dimethylethylenediamine (1.00 mL, 9.96 mmol) was added to a stirring solution of chloroform (20 mL) containing triethylamine (1.50 mL, 10.8 mmol) and di-*tert*-butyldicarbonate (2.5508 g, 11.7 mmol, Fluka, St. Louis, MO, USA). After 1 h, the reaction mixture was washed three times with aqueous 10% sodium bicarbonate (20 mL) before drying over MgSO_4_, filtering, and concentrating *in vacuo* to yield a clear oil. The resulting oil was resuspended in methanol (25 mL) and to this solution was added methyl iodide-*d_3_* (1.9352 g, 13.3 mmol, Cambridge Isotope Labs, Tewksbury, MA, USA), and KHCO_3_ (1.1286 g, 11.3 mmol). The mixture was stirred for 15 h, before concentrating under reduced pressure, resuspending in chloroform (50 mL), filtering and concentrating again to a clear oil. The Boc-protected cholamine-*d_3_* was precipitated by the addition of Et_2_O (70 mL). The supernatant was decanted, and the resulting salts were washed again with Et_2_O (40 mL) before drying *in vacuo* to give (2.6554 g, 80%) of Boc-protected cholamine-*d_3_* intermediate as the iodide salt. R_f_ = 0.79 (1:1:1:1 MeOH/H_2_O/BuOH/EtOAc); ^1^H-NMR (300 MHz; CDCl_3_; Me_4_Si) δ 5.45 (2 H, m), 4.13 (2 H, m), 3.55 (6 H, s), 3.46 (1 H, s), 1.48 (9 H, s); MS (HRESI-MS) calcd. for [C_10_H_20_D_3_N_2_O_2_]^+^ 206.1948, found 206.1952.

The Boc-protected cholamine-*d_3_* intermediate (2.6554 g, 7.97 mmol) was dissolved in methanol (20 mL) and cooled to 0 °C before the addition of freshly distilled acetyl chloride (2.5 mL, 35.2 mmol). The mixture was allowed to stir at 0 °C for 3 h before concentrating to a clear oil. The cholamine-*d_3_* was precipitated by the adddition of Et_2_O (50 mL) followed by decanting of the ether supernatant. The white solid was dried *in vacuo* to give (1.3643 g, 96%) of cholamine-*d_3_* as the chloide salt. R_f_ = 0.75 (1:1:1:1 MeOH/H_2_O/BuOH/EtOAc); ^1^H-NMR (300 MHz; CD_3_OD) δ 4.87 (3 H, s), 4.00 (2 H, m), 3.49 (2 H, m), 3.22 6 H, m); MS (HRESI-MS) calcd. for [C_5_H_12_D_3_N_2_]^+^ 106.1424, found 106.1430.

### 3.3. Labeling of Lauric Acid Standard with Cholamine

A lauric acid (Sigma-Aldrich) standard was prepared to 10 mM in dimethyl sulfoxide (DMSO). Three 50 µL aliquots were treated sequentially with the following: 125 µL of 20 mM 1-hydroxybenzotriazole (HOBt) in DMSO; 50 µL of 100 mM cholamine-*d_0_*, *d_3_*, or *d_9_* in DMSO containing 200 mM triethylamine (TEA, Aldrich); and 125 µL of 20 mM 2-(1H-benzotriazole-1-yl)-1,1,3,3-tetramethylaminium hexafluorophosphate (HBTU; Novabiochem, Gibbstown, NJ, USA) in DMSO. The samples were left to react overnight before being dissolved (1:75) in 3:1 water/acetonitrile with 0.1% formic acid. 15 µL from each of the three labeled samples (*d_0_*:d_3_:*d_9_*) were mixed 1:1:1. 2 μL of the combined labeling mixture was analyzed by HPLC-ESI-MS as outlined below.

### 3.4. Lipid Extraction from Egg Yolk

Following an established protocol, egg lipids were hydrolyzed and extracted from the egg yolks of commercially available eggs [37]. A 2:1 chloroform/methanol (12 mL) mixture was used to extract lipids from 3 grams of egg fat. A 1 mL aliquot of the extracted egg fat was placed into a 20 mL vial followed by 5 mL of 9:1 acetonitrile/5 M HCl. The solution was refluxed until hydrolysis was complete, identified by the complete dissolution of the oil layer. Samples were then dried on a rotary evaporator before the addition of 6 mL of dichloromethane and 2 mL of water. The organic layer was extracted and dried under reduced pressure.

### 3.5. Labeling of Chicken Egg Fatty Acids with Cholamine

Fatty acid extracts were dissolved in 5 µL of DMSO (~10 mM final concentration). 50 µL aliquots were treated sequentially with the following: 125 µL of 20 mM HOBt in DMSO; 50 µL of 100 mM cholamine-*d_0_*, *d_3_*, or *d_9_* in DMSO containing 200 mM TEA; and 125 µL of 20 mM HBTU in DMSO. The samples were left to react overnight before being dissolved (1:75) in 3:1 water/acetonitrile with 0.1% formic acid. 5 µL from each of the three labeled samples were mixed 1:1:1 unless otherwise stated. 2 μL of the combined labeling mixture was analyzed by HPLC-ESI-MS as outlined below. 

### 3.6. HPLC-ESI-MS/MS HCD Analysis

High energy collision dissociation (HCD) MS/MS analyses employed a capillary HPLC-ESI-MS/MS system consisting of a high performance liquid chromatograph (HPLC) (Waters nanoAcquity, Milford, MA, USA) connected to an electrospray ionization (ESI) FT/ion-trap mass spectrometer (LTQ Orbitrap Velos, Thermo Fisher Scientific, San Jose, CA, USA). A fritless 50 × 365 µm fused silica capillary micro-column was prepared by pulling the tip of the capillary to ~1 µm with a P-2000 laser puller (Sutter Instruments Co., Novato, CA, USA) and packing the capillary with 15 cm of 5 µm diameter C 18 beads (Western Analytical Products, Inc., Murrieta, CA, USA). A vented/trap column was prepared by packing 5 cm of the same C18 beads into a fritted 75 × 365 µm capillary. The fatty acids were loaded onto the trap column over 15 min at a flow-rate of 1 µL/min of 25% acetonitrile, 0.1% formic acid and eluted over 55 min at a flow-rate of 200 nL/min with a gradient of 25% to 98% acetonitrile in 0.1% formic acid. A full-mass scan was performed in the FT Orbitrap between 200–500 *m/z* at a resolution of 60,000, followed by ten MS/MS HCD scans of the ten highest intensity parent ions at 42% relative collision energy. The HCD scans were analyzed in the FT Orbitrap detector at a resolution of 7,500 and a mass range starting at 50 *m/z*.

## 4. Conclusions

This study demonstrated the use of a chemical derivatization approach to multiplexed analysis of fatty acids. Three isotopic variants of cholamine were synthesized and used to label, identify, and quantify fatty acid profiles in three chicken egg samples (caged- and cage free-derived) simultaneously. This multiplexed approach with cholamine labeling maintained the strong attributes of binary cholamine labeling including; pre-ionization, low limits of detection, chromatographic co-elution of labeled metabolites, and low coefficient’s of variation, all while increasing sample throughput. While this work demonstrates that a multiplexed approach to fatty acid analysis is feasible using a cholamine derivatization scheme, it also provides a methodology for multiplexed quantification of other carboxylic acid-containing metabolites. Metabolome sample throughput might be further improved by combining multiplex cholamine labeling with other functional group targeting chemical derivatization approaches.
